# Trends in Practice Patterns and Clinical Outcomes for Desmoid Tumors: A Large Single‐Institutional Australian Cohort

**DOI:** 10.1002/cam4.70973

**Published:** 2025-05-19

**Authors:** Joseph Sia, Stephanie Tan, Narayan Mohanakrishnan, Kelvin Yu, Samuel Y. Ngan, Sarat Chander, Jayesh Desai, Jeremy Lewin, Anne Hamilton, Stephen J. Luen, David E. Gyorki, Hayden Snow, Claudia DiBella, Sarah O'Reilly‐Harbidge, Lisa M. Orme, Julie Chu, Susie Bae

**Affiliations:** ^1^ Department of Radiation Oncology Peter MacCallum Cancer Centre Melbourne Australia; ^2^ Sir Peter MacCallum Department of Oncology University of Melbourne Melbourne Australia; ^3^ Department of Medical Oncology Peter MacCallum Cancer Centre Melbourne Australia; ^4^ Division of Cancer Surgery Peter MacCallum Cancer Centre Melbourne Australia

**Keywords:** active surveillance, aggressive fibromatosis, clinical study, desmoid tumors, patterns of practice, radiotherapy, surgery, targeted therapy

## Abstract

**Background:**

Desmoid tumors (DT) are rare, locally aggressive neoplasms that affect a young population and have a tendency for recurrence. There is sparse contemporary real‐world data to guide practice for DT. Here, we report on a large cohort of DT patients, describing patterns of care and clinical outcomes.

**Methods:**

Data on DT patients first seen between 2010 and 2021 were extracted from a prospective database and supplemented with a retrospective review of hospital records. Trends in treatment use were analyzed using the Cochran‐Armitage test. Time‐to‐next intervention (TTNI) was estimated with the Kaplan–Meier method. Imaging response was categorized using the RECIST v1.1 criteria.

**Results:**

A total of 135 patients, 265 treatment episodes were analyzed. Median follow‐up was 4.3 years. The common tumor sites were abdominal wall (27%), upper limb (20%), lower limb (16%), and intra‐abdominal (15%). Over time, the proportion of patients receiving no upfront treatment was stable (2010–2013: 31%, 2014–2017: 35%, 2018–2021: 29%; *p* = 0.5), but there was increasing first‐line use of NSAID/tamoxifen (7%, 41%, 47%; *p* < 0.001), and decreasing first‐line use of radiotherapy (35%, 14%, 4%; *p* < 0.001) and surgery (28%, 8%, 18%; *p* < 0.05). At 5 years, the proportion not requiring treatment switch was highest following surgery (72%), radiotherapy (66%), and no upfront therapy (52%). 12% and 5% of patients without treatment achieved partial and complete imaging responses at 2 years.

**Conclusion:**

We highlight the heterogeneity and trends in DT management over a 12‐year period, affirming the role of active surveillance, radiotherapy, and surgery in selected patients. Medical therapies are evolving and may significantly influence the DT management paradigm.

## 1 Introduction

Desmoid tumors (DT), also known as aggressive fibromatoses, are rare monoclonal fibroblastic tumors that can affect any part of the body, with an estimated yearly incidence of 2–4 new cases per million people [1]. Management of DTs is often a multi‐faceted discussion between the multi‐disciplinary medical teams and the patient. Because DTs typically affect young adults with an age peak of 30–40 years [2, 3], any intervention must consider long‐term or late effects that may manifest decades following therapy. Although DTs do not metastasize, they exhibit a variable clinical trajectory ranging from spontaneous regression to aggressive local recurrence and/or progression, which can be debilitating and even life‐threatening when involving vital structures. Therefore, careful patient selection for intervention is essential.

Treatment options for DTs include active surveillance (AS), surgery, radiation therapy (RT), non‐steroidal anti‐inflammatory drugs (NSAIDs), endocrine therapy, tyrosine kinase inhibitors, gamma secretase inhibitors, chemotherapy, and investigational approaches such as high intensity focused ultrasound, isolated limb perfusion, and cryoablation [4, 5, 6, 7, 8]. In recent decades, the management of DTs has evolved with a shift towards a more conservative approach. For asymptomatic patients, regardless of tumor site or size, guidelines from the international Desmoid Tumor Working Group (DTWG) in 2020 and 2024 [3, 9] advise a period of initial AS under a specialist team. Beyond this, the selection of the most appropriate therapy depends on factors including tumor symptoms and location, whether the tumor is associated with familial adenomatous polyposis (FAP) and patient preferences. Where surgical morbidity is limited and adequate surgical margins can be achieved, surgical resection as upfront therapy can be considered. Otherwise, RT offers excellent long‐term control, but this must be carefully balanced with the risk of late toxicities, such as fibrosis, functional impact, and secondary malignancies [10, 11, 12, 13]. Modern medical therapies have proven efficacy for DTs, though as a group they comprise a heterogenous range of agents. Recent consensus guidelines have deprioritized endocrine therapy and NSAIDs due to lack of efficacy, but highlighted active agents including tyrosine kinase inhibitors, gamma‐secretase inhibitors, and chemotherapy [3, 9]. Selection of these agents will be influenced by drug accessibility, tumor progression, symptoms, and patient tolerance and acceptance. FAP‐associated DTs tend to be more aggressive with multi‐focality and have higher morbidity and mortality compared to sporadic DTs, and hence this subpopulation of patients may benefit from more aggressive medical management [14].

Due to the rarity of DTs, there is a paucity of robust data comparing efficacy or informing optimal sequencing of treatment modalities. Clinicians often must extrapolate beyond the parameters of published reports to tailor treatment for the patient under their care, balancing achieving local control with treatment risks to achieve optimal long‐term health outcomes. Here, we report our experience in managing patients with DTs, where the vast majority of our patients with DTs were discussed in a multi‐disciplinary meeting. We analyzed trends in patterns of care and tumor control, and imaging response outcomes following various treatment strategies over a 12‐year period at both patient and treatment episode levels. These real‐world results from a large cohort add to the limited body of data for a rare disease with heterogeneous treatment strategies.

## 2 Materials and Methods

### 2.1 Patients

Approval by the Peter MacCallum Cancer Centre (PMCC)'s Human Research Ethics Committee was obtained for this study (QA/73959/PMCC). The Australian Comprehensive Cancer Outcomes and Research Database (ACCORD), a prospectively maintained database of all patients with bone and soft tissue tumors diagnoses seen at PMCC, was interrogated for eligible patients. Inclusion criteria were patients with DTs diagnosed between February 2010 and February 2021, who were managed at PMCC between February 2010 and September 2021. Those referred to PMCC for subsequent management with insufficient prior treatment details were excluded. A retrospective review of electronic medical records was performed to supplement this data. The presence of confirmed FAP was recorded for all patients. Performance status was graded using the Eastern Cooperative Oncology Group (ECOG) system. Tumor sizes were recorded based on the maximum tumor dimension on magnetic resonance imaging (MRI), computed tomography (CT), and/or ultrasound scans (USS) (in order of preference). Follow‐up data was collected until 31 July 2023.

### 2.2 Treatment Modalities and Definition of Endpoints

Treatment modalities were grouped as AS, NSAID/tamoxifen (patients in this group could have received NSAID, tamoxifen, or both), chemotherapy/targeted therapy/trial drug, RT, surgery, and focused ultrasound (US). Patients whose first line of active intervention was ≥ 6 months from diagnosis but did not have a formal documentation of AS were considered to have received a period of initial AS. AS was only recorded if it was used in the first line setting, that is, a period of subsequent observation following an active intervention was not considered AS.

Date of diagnosis was the date of procedure that provided histological confirmation. Follow‐up time was assessed from date of diagnosis. Treatment endpoints were time to next intervention (TTNI) and imaging response at 6‐, 12‐, and 24‐months post treatment, determined for each treatment episode for each patient. TTNI was defined as the time from diagnosis or start of an intervention to the start of a subsequent intervention. A TTNI event was defined as an intervention change due to progression in symptom or disease burden as judged by the treating clinician. A change in intervention due to other reasons, such as poor compliance or pre‐planned therapy switch (such as surgery following neoadjuvant RT) was considered a censored observation for that treatment episode.

Imaging response was categorized as complete response (CR), partial response (PR), stable disease (SD), or progressive disease (PD) using the Response Evaluation Criteria In Solid Tumors version 1.1 (RECIST) criteria, compared to the pre‐treatment baseline scan. In cases where tumors were not measurable by RECIST, an assessment was made based on the radiologist's impression from the imaging report. For a particular treatment episode, imaging responses were no longer collected upon change to the next line of therapy and were recorded as such. Instances where imaging was not done were excluded.

### 2.3 Statistical Analysis

Statistical comparison between groups was performed using the t and chi‐square tests for continuous and categorical variables, respectively. Trends in the change of treatment use were analyzed using the Cochran‐Armitage test. Median follow‐up time was assessed by the reverse Kaplan–Meier method. Time‐to‐next intervention (TTNI) was estimated with the Kaplan–Meier method and compared between subgroups using the log‐rank test.

## 3 Results

### 3.1 Baseline Characteristics

A total of 135 eligible patients and 265 treatment episodes were analyzed from 182 consecutive patients with DTs screened from ACCORD. The actuarial median follow‐up time was 4.3 years (IQR 2.5–6.7 years). Baseline patient and tumor characteristics for the overall cohort and stratified by first‐line (1L) interventions are outlined in Table 1. The median age was 36.7 years (range 14–76 years). Seventy percent (70%) of patients were female, 11% of whom were peripartum at the time of diagnosis. The most common histological diagnostic method was a core biopsy (81%), and 11 patients (8%) required a repeat biopsy to establish diagnosis. The most common tumor sites were torso (collectively 46%; in particular, abdominal wall in 27%), upper and lower limbs (collectively 36%; 20% and 16% respectively), and intra‐abdominal cavity (15%). Seven percent (7%) of patients had documented FAP syndrome. Antecedent trauma, including iatrogenic causes, was noted in 22% of patients. Four deaths occurred during the study period, one of which was DT‐related (sepsis from mesenteric DT in a patient with FAP, 11 months from diagnosis).

**TABLE 1:** Baseline patient and tumor characteristics (*n* = 135).

Characteristics	Overall	Active surveillance	Chemo/targeted/trial drug	NSAID/tamoxifen	RT	Surgery	*p*
	135	43	2	49	19	22	
Gender, *n* (%)							
Male	40 (29.6)	12 (27.9)	1 (50.0)	14 (28.6)	6 (31.6)	7 (31.8)	0.966
Female	95 (70.4)	31 (72.1)	1 (50.0)	35 (71.4)	13 (68.4)	15 (68.2)	
Age at diagnosis, years							
Median [interquartile range]	36.72 [28.65, 47.62]	35.27 [28.76, 42.96]	35.56 [29.53, 41.58]	37.63 [29.53, 48.70]	42.46 [27.02, 50.95]	36.76 [27.85, 45.17]	0.683
Performance status							
ECOG 0	115 (85.2)	37 (86.0)	2 (100.0)	41 (83.7)	14 (73.7)	21 (95.5)	0.368
ECOG 1	20 (14.8)	6 (14.0)	0 (0.0)	8 (16.3)	5 (26.3)	1 (4.5)	
Peripartum status							
No	85 (63.0)	24 (55.8)	1 (50.0)	33 (67.3)	13 (68.4)	14 (63.6)	0.422
Yes	10 (7.4)	7 (16.3)	0 (0.0)	2 (4.1)	0 (0.0)	1 (4.5)	
N/A	40 (29.6)	12 (27.9)	1 (50.0)	14 (28.6)	6 (31.6)	7 (31.8)	
Previous trauma at same site							
No	105 (77.8)	33 (76.7)	0 (0.0)	36 (73.5)	18 (94.7)	18 (81.8)	0.027
Yes	30 (22.2)	10 (23.3)	2 (100.0)	13 (26.5)	1 (5.3)	4 (18.2)	
FAP syndrome							
No	126 (93.3)	40 (93.0)	1 (50.0)	46 (93.9)	19 (100.0)	20 (90.9)	0.106
Yes	9 (6.7)	3 (7.0)	1 (50.0)	3 (6.1)	0 (0.0)	2 (9.1)	
Pain							
No	70 (51.9)	27 (63.8)	2 (100.0)	19 (38.8)	9 (47.4)	13 (59.1)	0.096
Yes	65 (48.1)	16 (37.2)	0 (0.0)	30 (61.2)	10 (52.6)	9 (40.9)	
Site							
Head and neck	3 (2.2)	2 (4.7)	0 (0.0)	0 (0.0)	1 (5.3)	0 (0.0)	0.014
Intra‐abdominal	21 (15.6)	5 (11.6)	1 (50.0)	7 (14.3)	0 (0.0)	7 (31.8)	
Intra‐thoracic	1 (0.7)	0 (0.0)	0 (0.0)	0 (0.0)	0 (0.0)	1 (4.5)	
Limb	49 (36.3)	11 (25.6)	0 (0.0)	21 (42.9)	13 (68.4)	4 (18.2)	
Torso	61 (45.2)	25 (58.1)	1 (50.0)	21 (42.9)	5 (26.3)	10 (45.5)	
Histological diagnosis method							
Core biopsy	109 (80.7)	38 (88.4)	2 (100.0)	46 (93.9)	15 (78.9)	8 (36.4)	< 0.001
Incisional biopsy	2 (1.5)	0 (0.0)	0 (0.0)	0 (0.0)	0 (0.0)	6 (27.3)	
Excisional biopsy	6 (4.4)	1 (2.3)	0 (0.0)	0 (0.0)	0 (0.0)	0 (0.0)	
Fine needle aspirate	1 (0.7)	1 (2.3)	0 (0.0)	0 (0.0)	0 (0.0)	1 (4.5)	
Unknown	17 (12.6)	3 (7.0)	0 (0.0)	3 (6.1)	4 (21.1)	7 (31.8)	
Tumor size							
Median [interquartile range]	57.0 [40.0, 84.25]	49.5 [31.8, 70.3]	200.00 [190.0, 210.0]	71.5 [45.5, 88.8]	56.0 [46.5, 92.5]	46.0 [39.0, 78.0]	0.002

*Note:* Interventions (columns) reflect first‐line treatments only.

More than two‐thirds of patients received AS or NSAID/tamoxifen as 1L intervention (31.9% and 36.3%, respectively) The 1L‐AS subgroup had the smallest mean tumor size (57 mm, range 18–181 mm), while the 1L‐chemotherapy/targeted/trial drug subgroup had the largest (200 mm, range 180–220 mm). There was a trend towards older age (median of 42.5 years versus 35.3–37.6 years) and limb DTs (68.4% vs. 0%–42.9%) in the 1L‐RT subgroup. No correlation was observed between the type of 1L intervention and patients who were pregnant or had FAP (*p* = 0.422 and *p* = 0.106, respectively). Surgical margins were involved in 68.6% (95% of which were non‐limb DTs), clear in 25.8%, and not reported/unknown in 5.6%. The most common RT dose‐fractionation schedules were 52–53.4 Gy (72.1%) and 50–50.4 Gy (23.3%) in 1.8–2 Gy per fraction. Across all treatment lines, a wide range of NSAIDs, tyrosine kinase inhibitors, and chemotherapy agents were used, which are outlined in Table S1.

### 3.2 Treatment Trends

**FIGURE 1:**
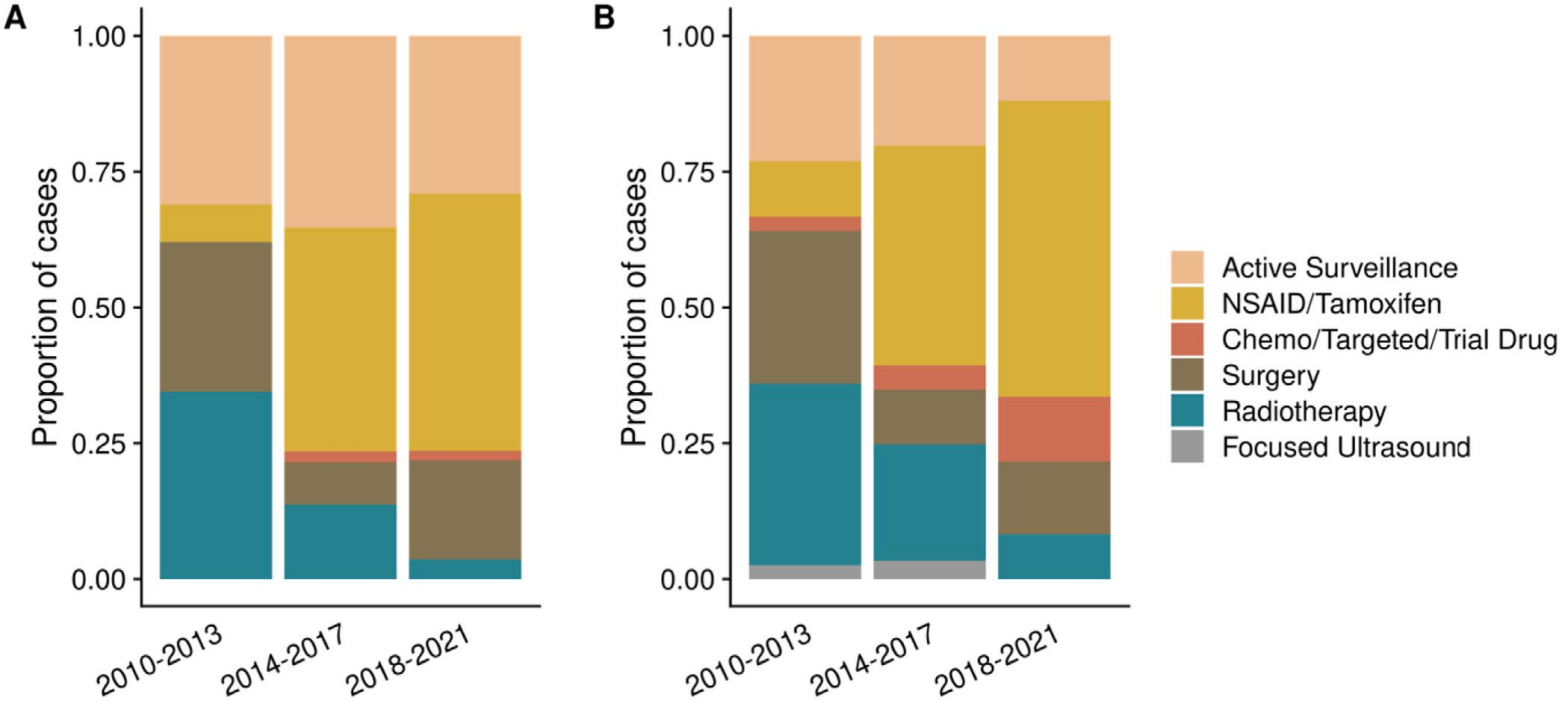
Treatment modality use over years, for (A) first‐line treatments only, and (B) all treatment lines. Active surveillance by definition can only be first‐line.

Trends in utilization of treatment types were assessed by dividing the 2010–2021 period into three 4‐year periods. For 1L interventions only, adoption of AS was stable across this time at around 32% (Figure 1A, Table S2). However, use of 1L‐NSAIDs/tamoxifen increased dramatically over this period, from 6.9% (2010–2013) to 47.3% (2018–2021). The inverse was observed for use of 1L‐RT, declining from 34.5% (2010–2013) to 3.6% (2018–2021). Interestingly, there was a dip in the proportion of intra‐abdominal tumors between 2014 and 2017 (7.8%, compared to 17.2% over 2010–2013 and 21.8% over 2018–2021), which may account for the drop in 1L‐surgery in the same period. Otherwise, there were no significant shifts in tumor site over time to confound this observation.

For all lines of intervention, a dominant trend of increasing NSAID/tamoxifen use was also observed, from 10.3% (2010–2013) to becoming the mainstay treatment strategy at 54.5% (2018–2021) (Figure 1B, Table S3). RT had the sharpest decrease in utilization from 33.3% (2010–2013) to 8.2% (2018–2021). Surgery use had a fall in proportion after the 2010–2013 period (28.2%) but remained stable thereafter (10.1%–13.4%).

### 3.3 Treatment Switch

**FIGURE 2:**
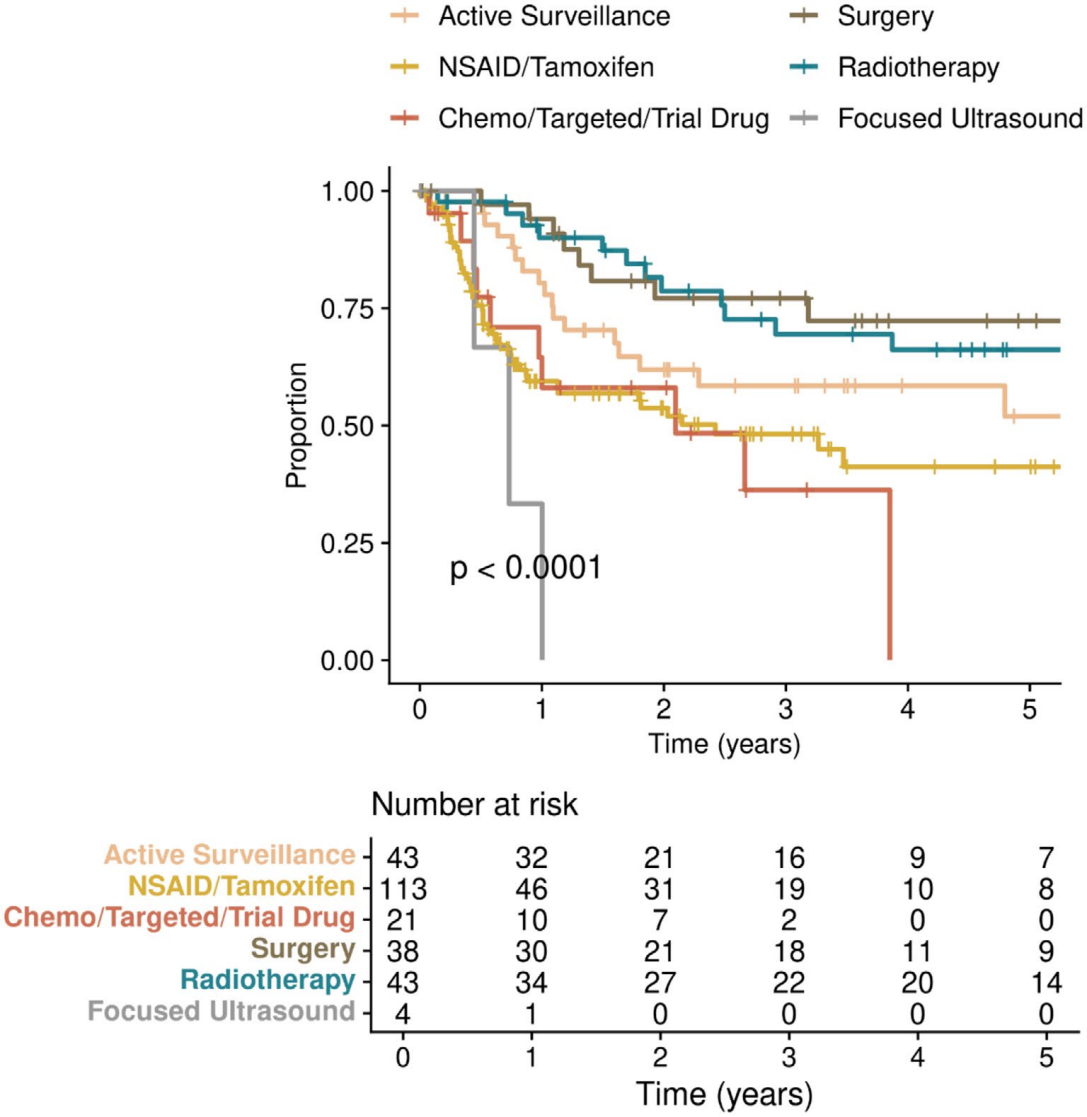
Time to next intervention (all lines of treatment).

Our TTNI definition (refer Section 2) serves as a surrogate for tumor control, as patients were not requiring a change in therapy. Surgery and RT conferred the highest TTNI with 77.1% and 81.6% of patients estimated to not require progression‐related treatment switch at 2 years, and 72.3% and 66.1% at 5 years, respectively (Figure 2). For AS and NSAID/tamoxifen, these rates were 61.9% and 53.7% at 2 years, and 52.0% and 41.2% at 5 years, respectively (Figure 2). For chemotherapy/targeted therapy/trial drug, 58.3% were estimated to not require a change in treatment at 2 years, and all patients in this subgroup had switched by 5 years (Figure 2). When restricting to the 1L setting only, the TTNI curves were not substantially different (Figure S1).

**FIGURE 3:**
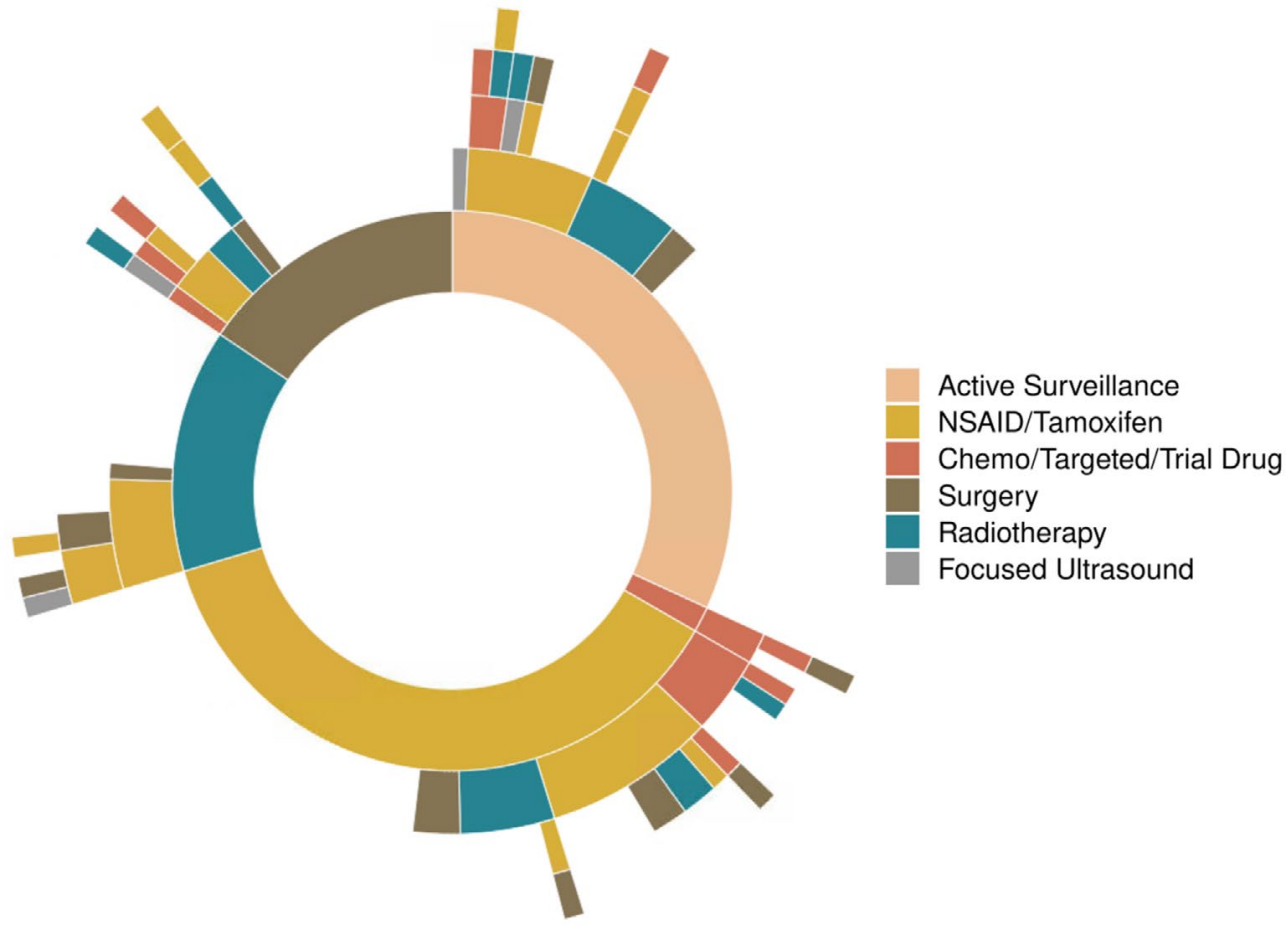
Intervention received by treatment line (inner circle represents first‐line).

Overall, the sequencing of treatment modalities after 1L intervention was highly variable (Figure 3). At the time of study analysis, approximately two‐thirds of patients undergoing AS, surgery, and RT had not required subsequent therapy (60%, 58% and 67%, respectively). One patient had planned pre‐operative RT followed by surgery for an upper limb tumor. No patients received adjuvant RT following surgery. For non‐AS interventions, TTNI was not different whether in first or subsequent‐line settings (*p* = 0.39).

Common reasons for stopping medical therapy were clinical futility as defined by clinician‐assessed disease or symptom progression (60.7%) and toxicity (25.0%) (Table S4). An issue with compliance was seen particularly for NSAIDs, accounting for 11–13% of cases. No RT‐related malignancies were observed in this study.

### 3.4 Imaging Response

**FIGURE 4:**
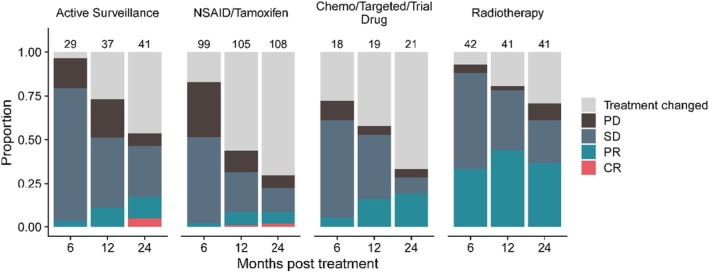
Imaging response at 6, 12, and 24 months for every treatment episode. Numbers on top of bars represent number of cases.

Imaging responses at 6, 12, and 24 months for every treatment episode, except for surgery (not relevant) and focused ultrasound (small numbers), are depicted in Figure 4. Notably, there was an increasing proportion of spontaneous partial and even complete responses over 24 months for the AS cohort, despite not having received therapeutic intervention (5% CR and 12% PR at 24 months, respectively). This should be borne in mind when interpreting responses in the other cohorts. Medical therapy primarily stabilized disease growth, while RT has a moderate chance of offering PR, but not CR (33%, 42% and 35% PR at 6, 12, and 24 months).

## 4 Discussion

This study reports on the largest cohort of patients and treatment episodes for DTs in Australia over a 12‐year contemporary period. Our results confirm that the conventional modalities of surgery and RT offer the advantage of tumor debulking or shrinkage (not just stabilization) on top of excellent long‐term control, though their use has been offset by a worldwide shift towards more conservative management with AS and NSAID/tamoxifen [3, 9]. The drop in the use of surgery particularly is reflected in global trends in patterns of practice [15, 16], though not universally [17]. Importantly, our data corroborates that AS is safe in appropriately selected patients [18, 19, 20], with 52% not requiring therapeutic intervention at 5 years. This is similar to other institution‐based studies, such as that by Rastrelli et al., which reported 44% of patients managed with AS not requiring change in management [21]. Consistent with other reports, 17% of DTs in our cohort underwent spontaneous regression at 2 years (5% CR) without any therapeutic intervention, and patients whose tumors remained controlled while on AS at 2–3 years were unlikely to require subsequent active intervention [19, 22, 23]. Nonetheless, tumors in the AS subgroup in our cohort were smaller than those in active intervention cohorts, and notably, 37% of these patients reported pain at baseline (Table 1). This practice is in contrast to the 2020 DTWG guidelines (published towards the end of this study period) which recommended AS based on an asymptomatic presentation, regardless of tumor size or site [3]. It will be interesting to observe if a shift in accordance with these guidelines will result in a difference in TTNI for the AS strategy.

The use of NSAID/tamoxifen increased significantly over the study period, while AS as 1L management remained steady. As an explanation for this trend, we postulate that a proportion of these patients may have been commenced on NSAIDs for pain relief, while still being effectively managed as AS. This is supported by the observation that 60% of patients who received 1L NSAID (±tamoxifen) reported pain as a symptom. The clinical burden of DTs is often high, with pain reported in up to 63% of patients who participated in a French Desmoid Advocacy group survey [24]. Another study by Penel et al. demonstrated that the presence of pain at baseline, compared to patients who were pain‐free, was associated with lower quality of life, higher functional impairment, and poorer event free survival [25]. Because these medical treatments are considered to have low toxicity, they could also have been commenced to manage patient (or even clinician) anxiety. In fact, a recent study considered NSAIDs and tamoxifen as ineffective for DT and categorized patients on these agents as under AS [18]. Furthermore, in that cohort (baseline symptoms not reported), the majority (69%) of patients were on NSAIDs, while only 25% were strictly not on any treatment. It is worth noting, however, that in our study the 1L‐AS and 1L‐NSAID/tamoxifen subgroups were not identical in regard to tumor size, site, and presenting symptoms (Table S1), which may explain the different TTNI curves between the two strategies (Figure 2). Aligned with international consensus, our unit has now moved away from using NSAID/tamoxifen as upfront therapy to AS in most patients and an earlier adoption of modern targeted therapies when treatment is required.

In this context, the group of chemotherapy, targeted therapy, and trial drugs has increased in use over time, reflecting the increased availability of effective medical therapy options. The recent FDA approval of nirogacestat in progressing DT based on the Phase 3 De‐Fi trial was an exciting new addition in the armamentarium of systemic therapies beyond cytotoxic chemotherapy and tyrosine kinase inhibitors such as sorafenib and pazopanib [6]. However, many questions remain regarding these therapies, including the optimal duration and sequencing of therapies. The possible long‐term toxicities of this class of drugs are also a concern in a disease that often afflicts a younger population. Superficially, it may appear surprising that the TTNI for these agents was only modest and not better than that of NSAID/tamoxifen, but tumors in this cohort were significantly larger at the time of treatment commencement, with many of the clinical trials using these agents requiring progressive disease at study entry. Furthermore, the limited cohort size hampers the confidence of this observation. This was due to difficult access to targeted therapy and trial drugs during the study period, with patients often requiring to self‐fund targeted therapies outside trials. Off‐label use of TKIs on compassionate access programs and a clinical trial involving a gamma secretase inhibitor (AL102) became an option at our institution only towards the end of this cohort period (Table S2); thus, this data does not reflect the more readily adopted use of these agents. While comparison with existing reports is highly challenging due to the wide range of agents used and small study numbers, our results fit within most studies of TKIs and chemotherapy agents, which report 12 month progression‐free survival rates generally between 60% and 80%, and overall response rates anywhere between 5% and 60% (being higher with cytotoxic chemotherapy) [3, 4, 19].

Selecting the most appropriate endpoint for assessment is challenging for DTs. Overall response rates are a helpful endpoint for medical therapies but not so much for surgery or RT. Progression‐free survival based on radiological evaluation is often used but is complicated by a reliance on regular imaging as well as the natural history of DTs, whereby tumors can have alternating intervals of progression, stability, and even regression. Furthermore, many DTs have complex radiological appearances that are not easily assessable by RECIST, hence creating a level of subjectivity [26]. Assessment of change in MRI T2 signal intensity is used in our center to detect treatment response, with T2 hyperintensity representing viable DT activity, but this evaluation is not yet standardized. Quality of life measures are highly important, but they are poorly documented in a consistent manner outside of clinical trials and can be difficult to analyze. In this study, we used a composite TTNI endpoint, for which an event is only triggered if the change in therapy is due to progressive disease or symptom burden. Otherwise, the therapy switch is considered a censored observation. Thus, a TTNI event in this study can be interpreted as progression due to the futility of the current treatment. This endpoint is objective, easily discerned from medical records, and meaningful for clinicians and patients.

Limitations of this study include caveats related to its retrospective and single‐institution nature. As alluded to above, given the progress in treatment options for DTs since our study period ended [9], our study population does not fully reflect the most contemporaneous practice in specialist centers, including the use of newer systemic therapies and locally ablative techniques such as cryoablation. Additionally, patients with DTs who were successfully managed with AS in the community were not fully captured in our database. For all patients, including those referred to PMCC after failure of AS in the community, the ACCORD database only recorded AS as an intervention if explicitly stated as such by the clinician. To address this, we automatically coded all patients who received their first active intervention ≥ 6 months from diagnosis as having received AS upfront, while also acknowledging this may not have captured all patients who were managed with initial AS intent. Another limitation is missing data on imaging responses due to a proportion of patients, especially those on AS, undergoing follow‐up imaging outside PMCC with no documentation in our system. The use of TTNI as an endpoint is therefore more reliable. Finally, TTNI outcomes in our study are not meant to be compared across treatment strategies as they are heavily influenced by patient selection. Ultimately, this study aims to provide a contemporary snapshot of the management of DTs in our institution to inform clinician and patient discussions using real‐world data, rather than to necessarily provide recommendations for clinical practice.

## 5 Conclusion

In this large real‐world cohort of DT patients and treatment episodes, we highlight the heterogeneity and changing trends in the management of DTs. AS, as recommended in DTWG guidelines, is a valid primary treatment for asymptomatic patients and is associated with a high rate of tumor control. RT and surgery remain effective treatments that offer excellent long‐term control of DTs in patients not suitable for AS. Importantly, the landscape of medical therapies, including new entries such as gamma secretase inhibitors, continues to evolve and has the potential to significantly shape the DT management paradigm. Similarly, the emergence of newer local ablative therapies, such as cryoablation, holds promise for treating DTs. Trials are ongoing to establish the optimal timing, sequencing, and its integration with medical therapies.

## Author Contributions

**Joseph Sia:** formal analysis (lead), investigation (equal), methodology (equal), supervision (equal), visualization (lead), writing – original draft (lead), writing – review and editing (lead). **Stephanie Tan:** data curation (lead), writing – original draft (equal), writing – review and editing (equal). **Narayan Mohanakrishnan:** data curation (equal). **Kelvin Yu:** data curation (equal), methodology (equal), supervision (equal). **Samuel Y. Ngan:** writing – review and editing (equal). **Sarat Chander:** writing – review and editing (equal). **Jayesh Desai:** writing – review and editing (equal). **Jeremy Lewin:** writing – review and editing (equal). **Anne Hamilton:** writing – review and editing (equal). **Stephen J. Luen:** writing – review and editing (equal). **David E. Gyorki:** writing – review and editing (equal). **Hayden Snow:** writing – review and editing (equal). **Claudia DiBella:** writing – review and editing (equal). **Sarah O'Reilly‐Harbidge:** writing – review and editing (equal). **Lisa M. Orme:** writing – review and editing (equal). **Julie Chu:** conceptualization (equal), methodology (equal), supervision (equal), writing – review and editing (equal). **Susie Bae:** conceptualization (equal), methodology (equal), supervision (equal), writing – review and editing (equal).

## Ethics Statement

Approval by the Peter MacCallum Cancer Centre (PMCC)'s Human Research Ethics Committee was obtained for this study, including a waiver of the requirement for consent (approval number QA/73959/PMCC).

## Conflicts of Interest

The authors declare no conflicts of interest.

## Supporting information


Data S1.

## Data Availability

The data that support the findings of this study are available from the corresponding author upon reasonable request.
