# Supplementary material for: Trends in Practice Patterns and Clinical Outcomes for Desmoid Tumors: A Large Single‐Institutional Australian Cohort
Source: Cancer Med. 2025 May 19;14(10):e70973. doi: 10.1002/cam4.70973 (PMC12086985; doi:10.1002/cam4.70973)
Supplement: Supplementary file 1 — Data S1. [file CAM4-14-e70973-s001.docx]

**Supplementary Table 1: Breakdown of medical therapy agents (all lines of treatment) (n=135)**

| **Medical therapy agent** | **n (%)** |
| --- | --- |
| Celecoxib | 48 (35.8) |
| Sulindac | 23 (17.2) |
| Sulindac and tamoxifen | 17 (12.7) |
| Tamoxifen | 15 (11.2) |
| Liposomal doxorubicin | 9 (6.7) |
| Celecoxib and tamoxifen | 8 (6.0) |
| Methotrexate and vinorelbine | 2 (1.5) |
| Pazopanib | 2 (1.5) |
| Sorafenib | 2 (1.5) |
| Sunitinib | 2 (1.5) |
| Trial drug (AL102 or placebo) | 2 (1.5) |
| Ibuprofen | 1 (0.7) |
| Naproxen | 1 (0.7) |
| Methotrexate and vinblastine | 1 (0.7) |
| Nirogacestat | 1 (0.7) |

**Supplementary Table 2: Treatment types grouped by time period (first-line treatments only)**

|  | Overall | 2010-2013 | 2014-2017 | 2018-2021 |
| --- | --- | --- | --- | --- |
| n (%) | 135 | 29 | 51 | 55 |
| Treatment type |  |  |  |  |
| Active surveillance | 43 (31.9) | 9 (31.0) | 18 (35.3) | 16 (29.1) |
| Chemo/Targeted/Trial Drug | 2 (1.5) | 0 (0.0) | 1 (2.0) | 1 (1.8) |
| NSAID/Tamoxifen | 49 (36.3) | 2 (6.9) | 21 (41.2) | 26 (47.3) |
| Radiotherapy | 19 (14.1) | 10 (34.5) | 7 (13.7) | 2 (3.6) |
| Surgery | 22 (16.3) | 8 (27.6) | 4 (7.8) | 10 (18.2) |

**Supplementary Table 3: Treatment types grouped by time period (all lines of treatment)**

|  | Overall | 2010-2013 | 2014-2017 | 2018-2021 |
| --- | --- | --- | --- | --- |
| n (%) | 262 | 39 | 89 | 134 |
| Treatment Type |  |  |  |  |
| Active surveillance | 43 (16.4) | 9 (23.1) | 18 (20.2) | 16 (11.9) |
| Chemo/Targeted/Trial Drug | 21 (8.0) | 1 (2.6) | 4 (4.5) | 16 (11.9) |
| Focused US | 4 (1.5) | 1 (2.6) | 3 (3.4) | 0 (0.0) |
| NSAID/Tamoxifen | 113 (43.1) | 4 (10.3) | 36 (40.4) | 73 (54.5) |
| Radiotherapy | 43 (16.4) | 13 (33.3) | 19 (21.3) | 11 (8.2) |
| Surgery | 38 (14.5) | 11 (28.2) | 9 (10.1) | 18 (13.4) |

**Supplementary Table 4: Reasons for cessation of medical therapy**

|  | Overall | Chemotherapy | NSAID | NSAID and Tamoxifen | Tamoxifen | Targeted therapy |
| --- | --- | --- | --- | --- | --- | --- |
| n | 84 | 8 | 45 | 19 | 9 | 3 |
| TTNI reason |  |  |  |  |  |  |
| Compliance | 8 (9.5) | 0 (0.0) | 6 (13.3) | 2 (10.5) | 0 (0.0) | 0 (0.0) |
| Clinical futility | 51 (60.7) | 6 (75.0) | 30 (66.7) | 9 (47.4) | 5 (55.6) | 1 (33.3) |
| Other | 3 (3.6) | 0 (0.0) | 1 (2.2) | 1 (5.3) | 1 (11.1) | 0 (0.0) |
| Pregnancy | 1 (1.2) | 0 (0.0) | 0 (0.0) | 0 (0.0) | 0 (0.0) | 1 (33.3) |
| Toxicity | 21 (25.0) | 2 (25.0) | 8 (17.8) | 7 (36.8) | 3 (33.3) | 1 (33.3) |

**Supplementary Figure 1: Time to next intervention (first-line treatments only)**

**
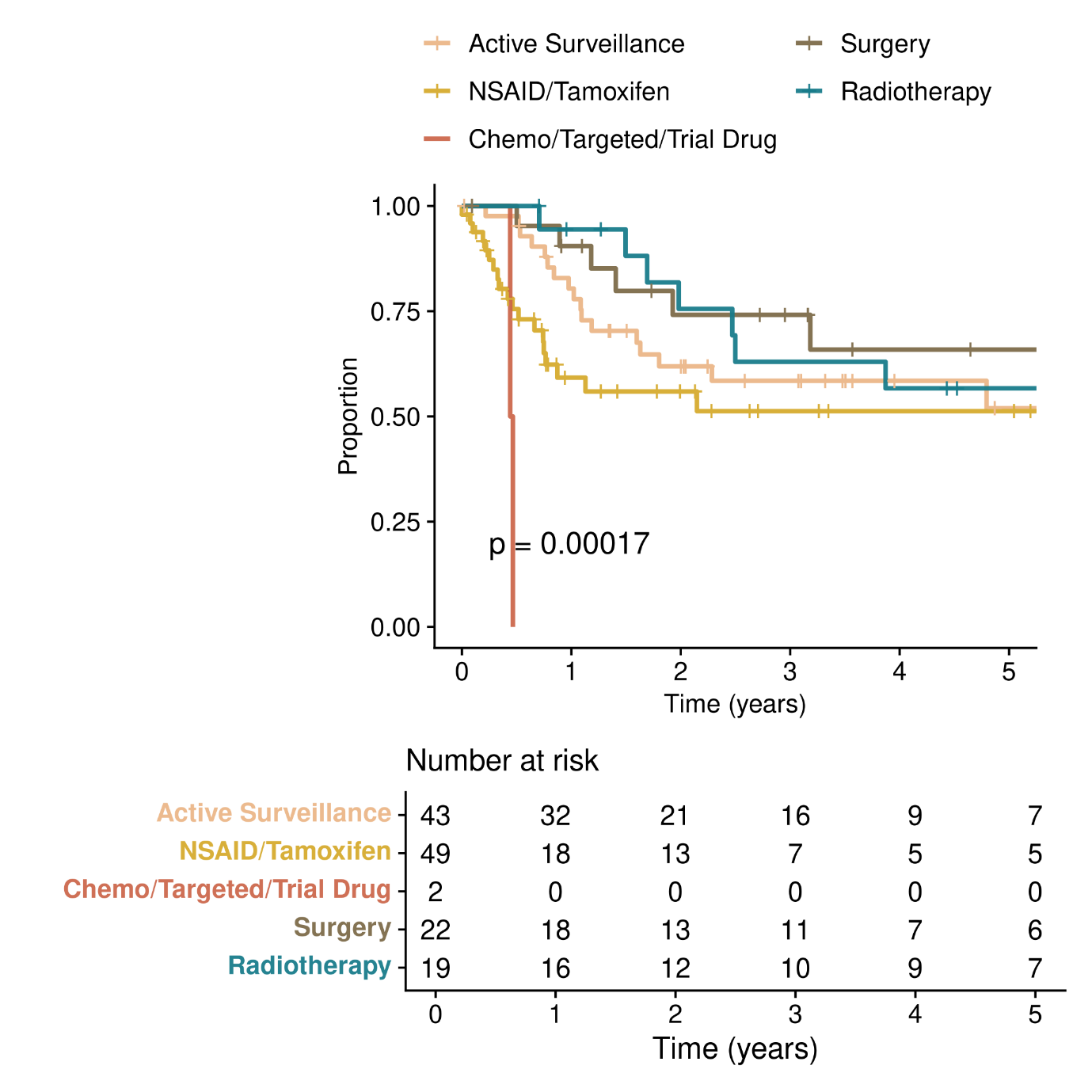
**
